# The Involvement of Hypothalamus–Pituitary–Adrenal (HPA) Axis in Suicide Risk

**DOI:** 10.3390/brainsci10090653

**Published:** 2020-09-21

**Authors:** Isabella Berardelli, Gianluca Serafini, Natalia Cortese, Federica Fiaschè, Rory C O’Connor, Maurizio Pompili

**Affiliations:** 1 Department of Neurosciences, Mental Health and Sensory Organs, Faculty of Medicine and Psychology, Suicide Prevention Center, Sant’Andrea Hospital, Sapienza University of Rome, 00100 Rome, Italy; isabella.berardelli@uniroma1.it (I.B.); cortesenatalia@yahoo.it (N.C.); fiaschefederica@libero.it (F.F.); 2 Department of Neuroscience, Rehabilitation, Ophthalmology, Genetics, Maternal and Child Health (DINOGMI), Section of Psychiatry, University of Genoa, IRCCS San Martino, Largo Rosanna Benzi 10, 16100 Genoa, Italy; gianluca.serafini@unige.it; 3 Suicidal Behaviour Research Laboratory, Institute of Health and Wellbeing, University of Glasgow, Glasgow G12 0XH, UK; Rory.OConnor@glasgow.ac.uk

**Keywords:** stress, Hypothalamic–Pituitary–Adrenal axis, suicidal behavior

## Abstract

Stress and Hypothalamic–Pituitary–Adrenal (HPA) axis dysregulation play a major role in various pathophysiological processes associated with both mood disorders and suicidal behavior. We conducted a systematic review with the primary aim of clarifying the nature and extent of HPA axis activity and suicidal behavior. The second aim of this review was to investigate whether potential biomarkers related to HPA axis abnormalities act as individual susceptibility factors for suicide. The PRISMA statement for reporting systematic reviews was used. Only articles published in English peer-reviewed journals were considered for possible inclusion; we excluded case reports, meta-analyses, and systematic reviews, and studies that did not clearly report statistical analysis, diagnostic criteria, or the number of patients included. Overall, 36 articles on HPA axis and suicide risk met inclusion criteria and were reviewed. Studies that investigated tests detecting biomarkers and the role of early life stressors in suicide risk were also included. We found that HPA axis activity is involved in suicide risk, regardless of the presence or absence of psychiatric conditions. The HPA axis abnormalities, mainly characterized by hyperactivity of the HPA axis, may exert an important modulatory influence on suicide risk. Impaired stress response mechanisms contribute to suicide risk. Targeting HPA axis dysregulation might represent a fruitful strategy for identifying new treatment targets and improving suicide risk prediction.

## 1. Introduction

**Figure 1:**
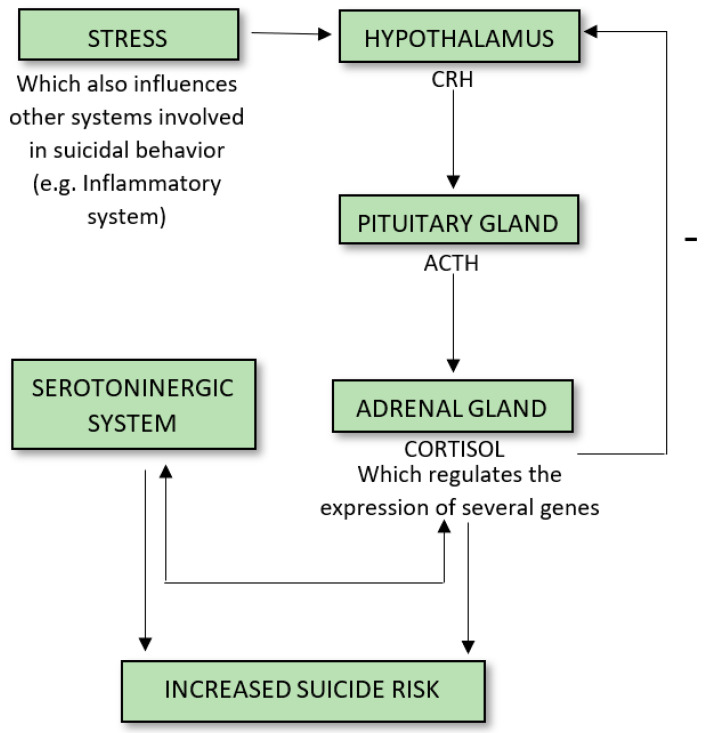
Hypothalamus–Pituitary–Adrenal (HPA) axis and suicide.

Given the prevalence of suicidal behavior in our society, preventing suicide should be a major health priority. Neuroimaging, genetic, and biochemical evidence support the existence of biological underpinnings related to suicidal behavior, implying the need for multidimensional preventive strategies against suicide [1,2]. There is an extensive body of evidence on risk factors, stressors, and populations at risk of suicide, and a number of theoretical models of suicide have been proposed in recent years [3,4]. These models include biological and psychodynamic theories of suicide, and cognitive and interpersonal theories (Interpersonal Theory of Suicide, Integrated motivational–volitional model, the Three-step theory 3ST, fluid vulnerability theory) [5]. One long-standing model [6] extended the diathesis-stress model to focus on the existence of a vulnerability or “diathesis” underlying suicidal behavior, which, in combination with different “stressors”, may result in actual suicidal behavior. A common feature of suicide risk models is that they tend to highlight the interaction between proximal and distal risk factors [7], including the complex and multifaceted relationship between external environment, life events, and suicide risk. There has also been a more recent focus on epigenetic alterations, which have mainly been related to maternal behavior, early life adversity, and in particular, a history of childhood sexual (CSA) and/or physical (CPA) abuse [8]. Furthermore, various lifetime stressors including traumatic events, experiences of loss and death, medical conditions, and physical and psychological pain are also established suicide vulnerability factors [9,10] (Figure 1). Acute and chronic stressors are both linked to complex physiological and behavioral responses. Specifically, the hypothalamic–pituitary–adrenal (HPA) axis is the neuroendocrine system that mainly regulates the body’s stress response, including modulating specific neurotransmitter levels in the brain. Neurons in the paraventricular nucleus (PVN) of the hypothalamus release a corticotropin-releasing factor (CRF) and arginine vasopressin (AVP), which stimulate the anterior pituitary gland and secrete an adrenocorticotropic hormone (ACTH). The ACTH, in turn, induces glucocorticoid synthesis. Hypothalamic activation of the HPA axis is modulated by several neurotransmitters with inhibitory (e.g., γ-aminobutyric acid (GABA) and opioids) and excitatory effects (e.g., norepinephrine and serotonin) on the PVN. To protect against prolonged activity, the HPA system is carefully modulated through negative-feedback loops designed to maintain predetermined hormone levels and homeostasis. Secretion of CRF, AVP, and ACTH in part are controlled by sensitive negative feedback exerted by cortisol at the level of the anterior pituitary gland, PVN, and hippocampus [11]. There are two types of receptors for cortisol—mineralocorticoid (type-I) and glucocorticoid (type-II) receptors—both of which are involved in the negative-feedback mechanisms. This negative feedback control mechanism maintains the secretion of ACTH and cortisol within a relatively narrow bandwidth. Growing evidence suggests that the FK506 binding protein 5 (FKBP5) regulates GR sensitivity [12].

Stress plays a major role in various pathophysiological processes associated with mood disorders and suicidal behavior [13]. More than 20 years of research has demonstrated that altered HPA axis regulation is involved in several psychiatric disorders [12]. Increased levels of stress are linked to several alterations in the HPA axis and dysregulation of the serotonergic system, involving 5-HT1A receptors in both individuals with mood disorders and those with a history of suicidal behavior, underling the importance of specific biomarkers in psychiatric conditions and in suicide risk, specifically [14]. However, the research literature suggests that HPA axis activity is involved in suicide risk, regardless of the presence or absence of psychiatric conditions.

The identification of specific biomarkers for the risk of psychiatric illness and suicide is important to guide the identification of preventive measures over time [9]. To this end, studies have demonstrated that HPA over-activity may be a good predictor of suicide risk, regardless of the existence of other psychiatric disorders [13]; however, the unmet need in suicidology is relevant, and the potential use of biomarkers for clinical management is of scientific and clinical interest. Although the HPA axis is known to play a fundamental role in suicidal behaviors, in particular in patients with affective disorders [15], the nature and extent of its involvement is still unclear.

In this paper, we performed a systematic review to comprehensively understand the role of the HPA axis dysregulation and the potential biomarkers related to HPA axis abnormalities, acting as individual susceptibility factors for suicide. For the present purpose, we reviewed studies that examined the involvement of CRH, cortical hypertrophy, CRF binding sites, and FK506-binding protein 51. We also examined studies on cortisol levels and studies testing the negative feedback system, as investigated by the dexamethasone suppression test (DST) and the Trier social stress test (TSSTa). Finally, we included studies on early life stress, HPA axis dysregulation, and HPA axis dysregulation tested in postmortem studies.

## 2. Methods

**Figure 2:**
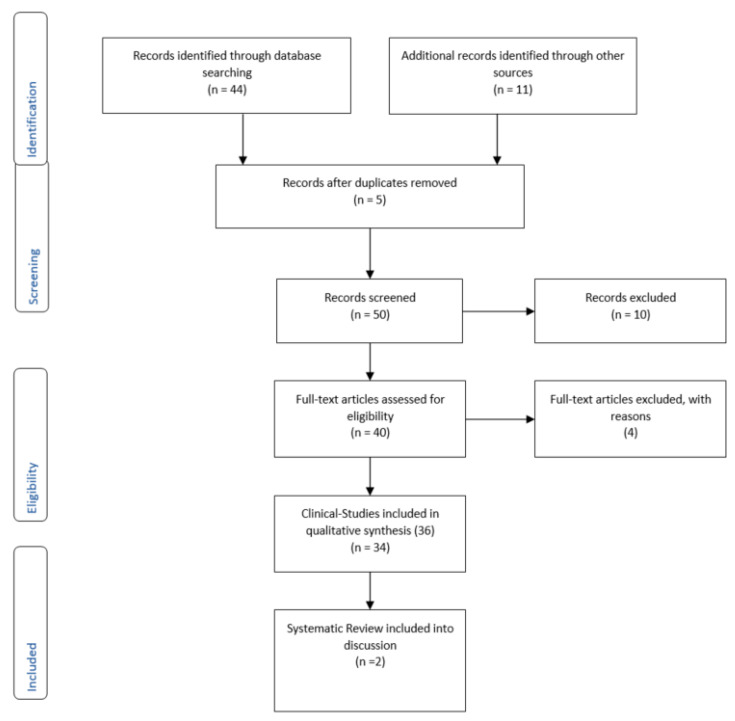
Flowchart of the search process for the systematic review.

We performed a systematic review of studies investigating the relationship between HPA axis dysregulation and suicidal risk. Six databases (MedLine, Excerpta Medica, PsycLit, PsycInfo, and Index Medicus) were searched to identify all papers about the main topic published between January 1980 and March 2020. The PRISMA statement for reporting systematic reviews was followed. The search terms which were used were: Hypothalamic–Pituitary–Adrenal axis OR Hypothalamic–Pituitary–Adrenal axis dysregulation OR Hypothalamic–Pituitary–Adrenal axis alteration OR HPA axis OR cortisol AND suicide risk OR suicide attempt OR suicide behavior OR suicide ideation. The titles and abstracts were reviewed initially and the selection criteria outlined above was applied. Only original articles on Hypothalamic–Pituitary–Adrenal axis alteration and suicide risk published in English language peer-reviewed journals were considered for inclusion, while all of the full-text articles were examined. In the results section, we excluded case reports, meta-analyses, and systematic reviews, and studies that did not clearly report statistical analysis, diagnostic criteria, or the numbers of patients included. Overall, we found 36 articles on HPA axis and suicide risk; of those articles, 11 investigated the role of CRH, cortical hypertrophy, CRF binding sites, and MHPG on suicide risk, 3 articles focused on the involvement of FK506-binding protein 51 (FKBP5/FKBP51), 17 articles reported tests on the function of HPA axis dysregulation, 4 studies on early life stress, HPA-axis dysregulation, and suicide as well as 2 post mortem studies (Figure 2).

The principal reviewer (IB) reviewed all the identified studies initially, and in addition, three reviewers independently inspected all the citations of the studies identified by searching them according to the main topic.

## 3. Results

### 3.1. CRH, Cortical Hypertrophy, CRF Binding Sites, and MHPG

The first studies that highlighted the role of HPA axis in suicidal behavior date back to 1980; several authors comparing depressed individuals who died by suicide to healthy controls reported elevated CRH and vasopressin levels in specific brain regions such as the forebrain, raphe, and locus ceruleus [16,17,18]. A positive correlation between adrenal weight and total cortical thickness in both left and right glands, probably due to cortical hypertrophy, was highlighted in patients who died by suicide [19,20,21]. Nemeroff and colleagues evaluated CRF binding sites in the frontal cortex of 26 suicide patients and 29 controls, demonstrating a significant reduction in the number of CRF binding sites (23%) of the frontal cortex of suicide patients [22]. In addition, Merali et al. [23], through PCR analyses in frontopolar cortex, highlighted a reduction of mRNA for CRH1 but not for CRH2 receptors, possibly secondary to the high levels of CRH activity. Lopez and coworkers [24] found evidence of chronic HPA axis activation through the pro-opiomelanocortin (POMC) mRNA, and a decrease in glucocorticoid receptor (GR) mRNA, in the anterior pituitaries of those who had died by suicide when compared to controls. Jokinen et al. [25], in a sample of 51 mood disorder inpatients, investigated the hypothesis that both 3-methoxy-4-hydroxphenylglycol (MHPG) in the cerebrospinal fluid (CSF) and HPA axis dysregulation were associated with suicidal behaviors. Results demonstrated that in nine individuals who had died by suicide, there was evidence of significantly lower CSF MHPG and baseline plasma cortisol than in people who have attempted suicide. The same research group [26], in a study of 88 individuals who had attempted suicide and deemed at high-risk or low-risk of suicide, investigated HPA axis-coupled CpG sites. These authors included CpG sites located within 2000 base pairs away from the transcriptional start site for CRH, corticotropin releasing hormone binding protein (CRHBP), corticotropin releasing hormone receptor 1 (CRHR1), corticotropin releasing hormone receptor 2 (CRHR2), FK506-binding protein 51 (FKBP5), and the glucocorticoid receptor (NR3C1). They found methylation shifts linked to the severity of suicide attempts among participants. Specifically, the methylation state of two corticotropin releasing hormone (CRH)-associated CpG sites were significantly hypomethylated in the high-risk group (*n* = 31), demonstrating epigenetic changes in the CRH gene related to severity of suicide attempts in adults and a general psychiatric risk in adolescents.

### 3.2. FK506-Binding Protein 51 (FKBP5/FKBP51)

Several studies focused on FK506-binding protein 51 (FKBP5/FKBP51) acting as co-chaperone modulates not only for glucocorticoid receptor activity in response to stressors, but also in a multitude of other cellular processes in both the brain and periphery [27]. Studies suggest that cortisol response to stress has a strong genetic etiology, and that the FK506 binding protein 5 (FKBP5) and the G-protein-coupled type-I CRH receptor (CRHR1) are prominent proteins regulating the stress response [27]. Variations in the genes encoding FKBP5 have been associated with several neuropsychiatric disorders. However, only a few studies have examined the association between FKBP5 and suicidal behavior [28]. In a Japanese population, haplotype analysis—a tool for ordering alleles on chromosomes—of the FKBP5 gene [29] suggested that haplotypes in the FKBP5 gene are associated with completed suicides. Specific studies analyzed the possible association between FKBP5 functional polymorphisms and completed suicide confirmed a significant association between the high-induction rs3800373 C allele and completed suicide [29]. The findings indicate that genetic alterations in FKBP5 might influence vulnerability to suicide [29].

### 3.3. Tests on the Function of the HPA Axis Dysregulation

Giletta et al. [30], in a study examining 138 adolescent females at risk of suicidal behaviors, investigated HPA axis stress responses by measuring cortisol levels pre and post stressors. Compared to controls, females in the hyperresponsive group were at an increased risk of reporting suicidal ideation 3 months later. Baseline salivary cortisol was also measured in another study of 69 patients with major affective disorders showing that those with a past suicide attempt history had lower baseline cortisol levels compared to those without a suicide attempt history [31].

#### 3.3.1. Dexamethasone Suppression Test (DST)

Available data concerning the existence of HPA axis dysregulation in suicidal behaviors are derived predominantly by studies examining the negative feedback system as tested by the dexamethasone suppression test (DST) (Table 1). In the study by Yerevanian et al. (2004) [32], depressed patients who were DST non-suppressors manifested a higher risk of suicide, presenting higher lethality attempts relative to DST suppressors. Moreover, Jokinen et al. [33] and (2008) [34] found that the dexamethasone suppression test may be a useful predictor of suicide. Additionally, in a second study, the same authors investigated the DST in 36 patients with mood disorders, with (*n* = 18) and without (*n* = 18) a history of suicide attempts; they found that the DST non-suppressor rate was 25% among mood disorder inpatients and 39% and 11% in those with and without a suicide attempt history, respectively [35]. Coryell and Schlesser [36], analyzing a cohort of patients with depression, highlighted that DST non-suppression at baseline was associated with a 14-fold higher odds of eventual suicide. Furthermore, Beauchaine and colleagues [37] demonstrated the association between lower post-DST cortisol, suicidal ideation, and self-inflicted injury SII, over and above parent reports. More recently, Ambrus et al. [38] investigated the relationship between leptin, HPA axis activity, and anxiety in sixty-nine individuals with a recent suicide attempt, and found that in females with a recent suicide attempt, lower CSF leptin levels were associated with anxiety symptoms and a hyperactive HPA axis.

#### 3.3.2. The Trier Social Stress Test and (TSSTa) and Maastricht Acute Stress Test (MAST)

The Trier social stress test (TSSTa) (Table 1) is a laboratory procedure used to reliably induce stress in human research participants used as an appropriate standardized protocol for studies of stress hormone reactivity. The TSST systematically induces a stress response in order to measure differences in reactivity and activation of the hypothalamic–pituitary–adrenal (HPA) axis during the task. The Maastricht Acute Stress Test (MAST), similarly to the TSST test, is a simple laboratory stress test capable of eliciting strong autonomic and glucocorticoid stress responses. Melhem et al. [39] examined cortisol responses to the Trier Social Stress Test (TSST) in 208 offspring of parents with mood disorders, demonstrating that offspring of those who had attempted suicide showed lower total cortisol output compared with offspring with suicide-related behavior (SRB) but who had never attempted suicide, non-suicidal offspring, and a healthy control group (Table 2). The same authors, Melhem et al. [40], confirmed previous results evaluating hair cortisol concentrations (HCC) in psychiatric patients at risk of suicide. Moreover, Eisenlohr-Moul et al. [41] measured cortisol responses to an adolescent modification of the TSST in a sample of 220 adolescent females and evaluated stress, suicidal ideation, and behaviors repeatedly across 18 months. Their findings suggested that peer stress acts as a trigger for suicidal ideation among female youths, but only among those with blunted cortisol reactivity [41]. Recently, Stanley and colleagues [42] hypothesized that different subgroups of those who have attempted suicide may differ in HPA axis dysregulation. They explored baseline cortisol, total cortisol output, and cortisol reactivity in participants who had both a mood disorder and a suicide attempt history (*N* = 35) and those with a suicide attempt history (*N* = 37) during the TSST. Specifically, those in the attempt group with higher impulsive aggression had a more pronounced cortisol response compared with other groups [42]. Additionally, Rizk and coworkers [43], in a sample of thirty-five individuals with major depressive disorder and 23 healthy controls, reported that individuals with brief, limited suicidal ideation (SI) are more stress-responsive (in the context of the TSST) than those with longer/continuous ideation. These authors reported that patients with brief periods of suicidal ideation exhibited greater cortisol response compared to those with longer/continuous ideation [43]. Shalev and colleagues [44] confirmed that the persistence of SI was associated with higher cortisol reactivity to stress, and that higher baseline cortisol levels may predict future SI (Table 2).

O’Connor et al. [45] investigated whether cortisol reactivity to the Maastricht Acute Stress Test (MAST) differs between patients with a previous suicide attempt, suicide ideators, and a control group. Results demonstrated that blunted HPA axis activity is associated with several suicidal behavior components. Specifically, patients who made a previous suicide attempt presented lower aggregate cortisol levels during the MAST compared to both the control group and patients with suicidal ideation.

### 3.4. Early Life Stress, HPA-Axis Dysregulation, and Suicide

It is well known that exposure to early life stress (ELS) predicts various psychiatric disorders [46]; however, the biological mechanisms accounting for this association are still unclear. Childhood physical, sexual, and emotional abuse and/or neglect are considered the most potent contributing risk factors for psychopathologies, that also increase suicide risk [46,47]. Early life traumatic experiences produce alterations in inflammation and HPA axis abnormalities, which increase vulnerability to stress [48,49]. Existing studies investigated the relationship between early traumatic experiences, HPA axis dysregulation and suicidal behavior [50] and several reports have demonstrated that childhood trauma is associated with HPA axis dysregulation and both factors increase the risk of suicidal behavior [51,52]. Roy et al. [53] hypothesized that corticotropin-releasing hormone high-affinity binding protein (CRHBP) variation and interaction with childhood trauma might influence suicidal behavior in individuals who have experienced childhood trauma. For instance, O’Connor and colleagues (2018) [54] investigated the association between childhood traumatic experiences, cortisol reactivity to a laboratory stressor, and resting cortisol levels in suicide risk patients and underlined that the highest levels of childhood traumatic events were reported in patients who had attempted suicide (78.7%), followed by those who had thought about suicide (37.7%) and then those with no suicidal history (17.8%). Results demonstrated that participants with a previous suicide attempt exhibited significantly lower aggregate cortisol levels compared to the control group, while participants who had attempted suicide and had a family history of suicide exhibited the lowest levels of cortisol in response to stress [53]. These results are consistent with other findings, indicating that blunted HPA axis activity is at least partially associated with suicidal behavior. Recently, O’Connor et al. [52] investigated the effects of childhood trauma, daily stress, and emotions on daily cortisol levels in individuals vulnerable to suicidal behavior and found that childhood trauma was associated with dysregulated cortisol reactivity to stress in adulthood. Sanabrais-Jiménez et al. [55], in a study of 366 patients with affective disorders (of which 183 were at risk of suicide), demonstrated an interaction between CRHR1 and CRHR2 with childhood trauma in patients with a history of suicidal acts, highlighting the relationship between the CRHR1 and CRHR2 genes with childhood trauma and suicide risk.

### 3.5. Post-Mortem Studies

The relationship between HPA axis dysregulation in those who have died by suicide as well as in those who have attempted suicide was also investigated in the postmortem brains of teenagers who had died by suicide. These analyses showed a decreased expression of glucocorticoid receptor (GR)-α and GR-inducible genes in the prefrontal cortex (PFC) and amygdala [56]. In another study [57], a decreased hippocampal GR expression was evident only among those who had died by suicide and who had experienced childhood abuse. How early life events may alter the expression of several hGR variants in the hippocampus of those who die by suicide through effects on promoter DNA methylation has been specifically investigated in this report, underling the relation between early life events and the expression of several hGR variants in the hippocampus of suicide patients.

## 4. Discussion

The main findings reviewed in the present paper, taken as a whole, suggest that HPA axis activity is involved in increased suicide risk, regardless of the presence or absence of psychiatric conditions.

Studies on CRH, cortical hypertrophy, CRF binding sites and MHPC suggest that HPA axis activity is involved in suicide risk, regardless of the presence or absence of psychiatric conditions. Several studies stressed the role of the HPA axis in suicide risk by assessing the levels of CRH and vasopressin in the forebrain, raphe, and locus ceruleus [15,16,17]; other studies have evaluated adrenal weight and total cortical thickness in suicide victims [18,19,20] as well as CRF binding sites and mRNA for CRH1, but not for CRH2 receptors [21,22]. Studies on haplotypes in the FKBP5 gene which modulates glucocorticoid receptor activity in response to stressors also suggested an association with suicide deaths [27,28,29].

Most data implicating the HPA axis come from studies examining the negative feedback system with the dexamethasone suppression test (DST) and TSSTa. The dexamethasone suppression tests showed a dysregulation of the HPA axis in participants with suicidal behaviors and the Trier Social Stress showed that patients who had attempted suicide displayed a lower total cortisol output. A limited number of post-mortem studies in patients who have died by suicide showed a decreased expression of glucocorticoid receptors.

Finally, an important finding is that several studies showed that participants with early life traumatic experiences produce alterations in HPA axis and have an increase in risk of suicidal behavior. Although the literature demonstrates an important role of childhood maltreatment in suicide risk [56], the mechanisms underlying this association are not yet completely understood and several theories are proposed. However, the results of our review demonstrate that, among the various biological, psychological, and social factors that increase suicidal risk in childhood with early life stress events, alterations in the HPA axis seem to play a relevant role.

Studies investigating the association between HPA axis and suicide risk are, however, still limited and it is still unclear whether the association between HPA axis and suicide risk is related to the presence or absence of psychiatric conditions [58]. Despite these limitations, the findings presented in this review highlight that impaired stress response mechanisms contribute significantly to suicide risk, and increased activity of stress response to early life adversities may exert deleterious effects on the development of brain structures implicated in suicidal behavior. In both of these contexts, genes may contribute to altered neurobiological functions. Studies that include an examination of early life stress, markers of biological function, and/or intermediate phenotypes of suicidal behavior are needed to shed further light on the complexities of the relationship between stress, genes, and suicidal behavior.

The importance of studying the involvement of the HPA axis in suicide risk is also based on the observation that the HPA is involved with several brain neurotransmitters, including serotonin, noradrenaline, and dopamine, all of which are involved in the neurobiology of suicide risk. Several studies have underlined that the depletion of noradrenaline and the decrease in serotonergic function in patients at risk of suicide and an increase in dopamine metabolism after exposure to stress, might have important physiological consequences. In particular, the HPA axis has a bidirectional relationship with the serotonergic system involving the amygdala, raphe nuclei, locus coeruleus (LC), and the hippocampus, and in modulating serotonergic neurotransmission directly regulating 5-HT receptors [59]. Furthermore, noradrenergic overactivity due to an overactivity of the HPA axis has been associated with higher suicide risk and endocrine responses to stress involves the medial prefrontal cortex (mPFC) and modulation of the subcortical pathways in which dopamine is mainly involved [60].

In conclusion, the HPA axis may exert an important modulatory influence on suicide risk and this dysregulation is associated with other systems implicated in suicide, including serotonin, opioids, glutamate systems, inflammatory pathways, lipid status, and neuroplasticity or neurogenesis. Suicide risk characterized by a range of different biomarkers, rather than a single biological dysregulation system, is central to the identification of new treatment targets and improvements in suicide risk prediction.

## Figures and Tables

**Table 1:** Dexamethasone suppression test and suicide risk.

Author, Year	Sample	Method	Results
*Giletta* et al. 2015 [30]	138 adolescent females at risk for SB	PST	The hyperresponsive group was more likely to report a lifetime history of SI
*Jokinen* et al. 2007 [33]	382 psychiatric inpatients with mood disorder	DST	Non-suppressor status was significantly associated with suicide
*Jokinen* et al. 2008 [34]	99 depressed elderly inpatients	DST	DST non-suppression distinguished between suicides and survivors in this sample
*Jokinen* et al. 2009 [35]	36 patients with mood disorder, with (*n* = 18) and without suicide attempt	DST	DST non-suppressor rate was 39% in those with a suicide attempt history and 11% in those with a non-attempt history
*Coryell* and *Schlesser* 2001 [36]	78 patients with mood disorder	DST	Estimated risk for suicide in the group with abnormal DST results was 26.8%, compared to only 2.9% for the other group
*Beauchaine* et al. 2015 [37]	57 adolescent females (13–17 years)	DST	Lower post-DST cortisol was associated with suicidal ideation and self-injury

SB: Suicidal Behavior. SA: Suicidal Attempters. MDD: Major Depressive Disorder. DST: Dexametasome Suppression Test. SI: Suicidal Ideation.

**Table 2:** Trier Social Stress Test and suicide risk and the Maastricht Acute Stress Test.

Author, Year	Sample	Method	Results
*Melhem* et al. 2016 [40]	208 offspring of parents with mood disorder	TSST	Offspring with a suicide attempt history showed lower total cortisol output
*Eisenlohr-Moul* et al. 2018 [41]	220 adolescent females	TSST	Stress triggers suicidal behavior among those with blunted cortisol reactivity
*Stanley* et al. 2019 [42]	72 patients with mood disorder (SA *n* = 35, non-SA *n* = 37)	TSST	Stress response differed based on impulsivity/aggression levels in those who had attempted suicide (with more pronounced cortisol response)
*Rizk* et al. 2018 [43]	35 patients with MDD, 23 healthy controls	TSST	Patients with brief suicidal ideation presented greater cortisol response
*Shalev* et al. 2019 [44]	114 youth bereaved by sudden parental death, 109 non-bereaved controls	TSST	Cortisol reactivity to stress was higher in those with high SI trajectory
*O’Connor* et al. 2017 [45]	145 patients (47 attempter, 53 with SI, 45 control group)	MAST	HPA axis activity is markedly lower in suicide attempters compared to controls, but not ideators

SB: Suicidal Behavior. SA: Suicidal Attempters. MDD: Major Depressive Disorder. TSST: Trier Social Stress Test. SI: Suicidal Ideation. MAST: Maastricht Acute Stress Test.

## References

[1] Turecki G., Brent D.A. (2016). Suicide and suicidal behaviour. Lancet.

[2] Pompili M., Gibiino S., Innamorati M., Serafini G., Del Casale A., De Risio L., Palermo M., Montebovi F., Campi S., De Luca V. (2012). Prolactin and thyroid hormone levels are associated with suicide attempts in psychiatric patients. Psychiatry Res..

[3] Turecki G., Brent D.A., Gunnell D., O’Connor R.C., Oquendo M.A., Pirkis J., Stanley B.H. (2019). Suicide and suicide risk. Nat. Rev. Dis. Primers.

[4] O’Connor R.C., Kirtley O.J. (2018). The Integrated Motivational-Volitional Model of Suicidal Behaviour. Philos. Trans. R. Soc. B.

[5] Selby E.A., Joiner T.E., Ribeiro J., Matthew K. (2014). Comprehensive Theories of Suicidal Behaviors. The Oxford Handbook of Suicide and Self-Injury.

[6] Mann J.J., Waternaux C., Haas G.L., Malone K.M. (1999). Toward a clinical model of suicidal behavior in psychiatric patients. Am. J. Psychiatry.

[7] Hawton K., Van Heeringen K. (2009). Suicide. Lancet.

[8] Labonte B., Turecki G. (2010). The epigenetics of suicide: Explaining the biological effects of early life environmental adversity. Arch. Suicide Res..

[9] Lutz P.E., Mechawar N., Turecki G. (2017). Neuropathology of suicide: Recent findings and future directions. Mol. Psychiatry.

[10] Pompili M., Shrivastava A., Serafini G., Innamorati M., Milelli M., Erbuto D., Ricci F., Lamis D.A., Scocco P., Amore M. (2013). Bereavement after the suicide of a significant other. Indian J. Psychiatry.

[11] McEwen B.S. (2007). Physiology and neurobiology of stress and adaptation: Central role of the brain. Physiol. Rev..

[12] Dedovic K., Duchesne A., Andrews J., Engert V., Pruessner J.C. (2009). The brain and the stress axis: The neural correlates of cortisol regulation in response to stress. Neuroimage.

[13] Ventriglio A., Gentile A., Baldessarini R.J., Bellomo A. (2015). Early-life stress and psychiatric disorders: Epidemiology, neurobiology and innovative pharmacological targets. Curr. Pharm. Des..

[14] Sudol K., Mann J.J. (2017). Biomarkers of Suicide Attempt Behavior: Towards a Biological Model of Risk. Curr. Psychiatry Rep..

[15] Menke A. (2019). Is the HPA Axis as Target for Depression Outdated, or Is There a New Hope?. Front. Psychiatry.

[16] Merali Z., Du L., Hrdina P., Palkovits M., Faludi G., Poulter M.O., Anisman H. (2004). Dysregulation in the suicide brain: mRNA expression of corticotropin-releasing hormone receptors and GABA (A) receptor subunits in frontal cortical brain region. J. Neurosci..

[17] Austin M.C., Janosky J.E., Murphy H.A. (2003). Increased corticotropin-releasing hormone immunoreactivity in monoamine-containing pontine nuclei of depressed suicide men. Mol. Psychiatry.

[18] Arató M., Bánki C.M., Bissette G., Nemeroff C.B. (1989). Elevated CSF CRF in suicide victims. Biol. Psychiatry.

[19] Dumser T., Barocka A., Schubert E. (1998). Weight of adrenal glands may be increased in persons who commit suicide. Am. J. Forensic Med. Pathol..

[20] Szigethy E., Conwell Y., Forbes N.T., Cox C., Caine E.D. (1994). Adrenal weight and morphology in victims of completed suicide. Biol. Psychiatry.

[21] Stein E., McCrank E., Schaefer B., Goyer R. (1993). Adrenal gland weight and suicide. Can. J. Psychiatry.

[22] Nemeroff C.B., Owens M.J., Bissette G., Andorn A.C., Stanley M. (1988). Reduced corticotropin releasing factor binding sites in the frontal cortex of suicide victims. Arch. Gen. Psychiatry.

[23] Merali Z., Kent P., Du L., Hrdina P., Palkovits M., Faludi G., Poulter M.O., Bédard T., Anisman H. (2006). Corticotropin-releasing hormone, arginine vasopressin, gastrin-releasing peptide, and neuromedin B alterations in stress-relevant brain regions of suicides and control subjects. Biol. Psychiatry.

[24] López J.F., Palkovits M., Arató M., Mansour A., Akil. H., Watson S.J. (1992). Localization and quantification of pro-opiomelanocortin mRNA and glucocorticoid receptor mRNA in pituitaries of suicide victims. Neuroendocrinology.

[25] Jokinen J., Ouda J., Nordström P. (2010). Noradrenergic function and HPA axis dysregulation in suicidal behaviour. Psychoneuroendocrinology.

[26] Jokinen J., Boström A.E., Dadfar A., Ciuculete D.M., Chatzittofis A., Asberg M., Schioth H.B. (2018). Epigenetic Changes in the CRH Gene are Related to Severity of Suicide Attempt and a General Psychiatric Risk Score in Adolescents. EBioMedicine.

[27] Cioffi D.L., Hubler T.R., Scammell J.G. (2011). Organization and function of the FKBP52 and FKBP51 genes. Curr. Opin. Pharmacol..

[28] Fudalej S., Kopera M., Wołyńczyk-Gmaj D., Fudalej M., Krajewski P., Wasilewska K., Szymański K., Chojnicka I. (2015). Association between FKBP5 Functional Polymorphisms and Completed Suicide. Neuropsychobiology.

[29] Supriyanto I., Sasada T., Fukutake M., Asano M., Ueno Y., Nagasaki Y., Shirakawa O., Hishimoto A. (2011). Association of FKBP5 gene haplotypes with completed suicide in the Japanese population. Prog. Neuropsychopharm. Biol. Psychiatry.

[30] Giletta M., Calhoun C.D., Hastings P.D., Rudolph K.D., Nock M.K., Prinstein M.J. (2015). Multi-Level Risk Factors for Suicidal Ideation Among at-Risk Adolescent Females: The Role of Hypothalamic-Pituitary-Adrenal Axis Responses to Stress. J. Abnorm. Child Psychol..

[31] Keilp J.G., Stanley B.H., Burke A.K., Melhem N.M., Oquendo M.A., Brent D.A., Mann J.J. (2016). Further evidence of low baseline cortisol levels in suicide attempters. J. Affect. Disord..

[32] Yerevanian B.I., Feusner J.D., Koek R.J., Mintz J. (2004). The dexamethasone suppression test as a predictor of suicidal behavior in unipolar depression. J. Affect. Disord..

[33] Jokinen J., Carlborg A., Martensson B., Forslund K., Nordström A.L., Nordström P. (2007). DST non-suppression predicts suicide after attempted suicide. Psychiatry Res..

[34] Jokinen J., Nordström P. (2008). HPA axis hyperactivity as suicide predictor in elderly mood disorder inpatients. Psychoneuroendocrinology.

[35] Jokinen J., Nordström P. (2009). HPA axis hyperactivity and attempted suicide in young adult mood disorder inpatients. J. Affect. Disord..

[36] Coryell W., Schlesser M. (2001). The dexamethasone suppression test and suicide prediction. Am. J. Psychiatry.

[37] Beauchaine T.P., Crowell S.E., Hsiao R.C. (2015). Post-dexamethasone cortisol, self-inflicted injury, and suicidal ideation among depressed adolescent girls. J. Abnorm. Child Psychol..

[38] Ambrus L., Westling S. (2019). Leptin, Anxiety Symptoms, and Hypothalamic-Pituitary-Adrenal Axis Activity among Drug-Free, Female Suicide Attempters. Neuropsychobiology.

[39] Melhem N.M., Keilp J.G., Porta G., Oquendo M.A., Burke A., Stanley B., Cooper T.B., Mann J.J., Brent D.A. (2016). Blunted HPA Axis Activity in Suicide Attempters Compared to those at High Risk for Suicidal Behavior. Neuropsychopharmacology.

[40] Melhem N.M., Munroe S., Marsland A., Gray K., Brent D., Porta G., Douaihy A., Laudenslager M.L., DePietro F., Diler R. (2017). Blunted HPA axis activity prior to suicide attempt and increased inflammation in attempters. Psychoneuroendocrinology.

[41] Eisenlohr-Moul T.A., Miller A.B., Giletta M., Hastings P.D., Rudolph K.D., Nock M.K., Prinstein M.J. (2018). HPA axis response and psychosocial stress as interactive predictors of suicidal ideation and behavior in adolescent females: A multilevel diathesis-stress framework. Neuropsychopharmacology.

[42] Stanley B., Michel C.A., Galfalvy H.C., Keilp J.G., Rizk M.M., Richardson-Vejlgaard R., Oquendo M.A., Mann J.J. (2019). Suicidal subtypes, stress responsivity and impulsive aggression. Psychiatry Res..

[43] Rizk M.M., Galfalvy H., Singh T., Keilp J.G., Sublette M.E., Oquendo M.A., Mann J.J., Stanley B. (2018). Toward subtyping of suicidality: Brief suicidal ideation is associated with greater stress response. J. Affect. Disord..

[44] Shalev A., Porta G., Biernesser C., Zelazny J., Walker-Payne M., Melhem N., Brent D. (2019). Cortisol response to stress as a predictor for suicidal ideation in youth. J. Affect. Disord..

[45] O’Connor D.B., Green J.A., Ferguson E., O’Carroll R.E., O’Connor R.C. (2017). Cortisol reactivity and suicidal behavior: Investigating the role of hypothalamic-pituitary-adrenal axis responses to stress in suicide attempters and ideators. Psychoneuroendocrinology.

[46] McCrory E., De Brito S.A., Viding E. (2012). The link between child abuse and psychopathology: A review of neurobiological and genetic research. J. R. Soc. Med..

[47] Jaffee S.R. (2017). Child Maltreatment and Risk for Psychopathology in Childhood and Adulthood. Annu. Rev. Clin. Psychol..

[48] Green J.G., Mclaughlin K.A., Berglund P.A., Gruber M.J., Sampson N.A., Zaslavsky A.M., Kessler R.C. (2010). Childhood Adversities and Adult Psychiatric Disorders in the National Comorbidity Survey Replication I. Arch. Gen. Psychiatry.

[49] Heim C., Shugart M., Shugart M., Craighead W.E., Nemeroff C.B. (2010). Neurobiological and psychiatric consequences of child abuse and neglect. Dev. Psychobiol..

[50] Heim C., Newport D.J., Mletzko T.M., Bonsall R., Miller A.H., Nemeroff C.B. (2008). The link between childhood trauma and depression: Insights from HPA axis studies in humans. Psychoneuroendocrinology.

[51] McGowan P.O., Sasaki A., D’Alessio A.C., Dymov S., Labonté B., Szyf M., Turecki G., Meaney M.J. (2009). Epigenetic regulation of the glucocorticoid receptor in human brain associates with childhood abuse. Nat. Neurosci..

[52] O’Connor D.B., Branley-Bell D., Green J.A., Ferguson E., O’Carroll R.E., O’Connor R.C. (2020). Effects of childhood trauma, daily stress, and emotions on daily cortisol levels in individuals vulnerable to suicide. J. Abnorm. Psychol..

[53] Roy A., Hodgkinson C.A., Deluca V., Goldman D., Enoch M.A. (2012). Two HPA axis genes, CRHBP and FKBP5, interact with childhood trauma to increase the risk for suicidal behavior. J. Psychiatr. Res..

[54] O’Connor D.B., Green J.A., Ferguson E., O’Carroll R.E., O’Connor R.C. (2018). Effects of childhood trauma on cortisol levels in suicide attempters and ideators. Psychoneuroendocrinology.

[55] Sanabrais-Jiménez M.A., Sotelo-Ramirez C.E., Ordoñez-Martinez B., Jiménez-Pavón J., Ahumada-Curiel G., Piana-Diaz S., Flores-Flores G., Flores-Ramos M., Jiménez-Anguiano A., Camarena B. (2019). Effect of CRHR1 and CRHR2 gene polymorphisms and childhood trauma in suicide attempt. J. Neural. Transm..

[56] Pandey G.N., Rizavi H.S., Ren X., Dwivedi Y., Palkovits M. (2013). Region-specific alterations in glucocorticoid receptor expression in the postmortem brain of teenage suicide victims. Psychoneuroendocrinology.

[57] Labonte B., Yerko V., Gross J., Mechawar N., Meaney M.J., Szyf M., Turecki G. (2012). Differential glucocorticoid receptor exon 1(B), 1(C), and 1(H) expression and methylation in suicide completers with a history of childhood abuse. Biol. Psychiatry.

[58] Janiri D., De Rossi P., Kotzalidis G.D., Girardi P., Koukopoulos A.E., Reginaldi D., Dotto F., Manfredi G., Jollant F., Gorwood P. (2018). Psychopathological characteristics and adverse childhood events are differentially associated with suicidal ideation and suicidal acts in mood disorders. Eur. Psychiatry.

[59] Pompili M., Serafini G., Innamorati M., Möller-Leimkühler A.M., Giupponi G., Girardi P., Tatarelli R., Lester D. (2010). The hypothalamic-pituitary-adrenal axis and serotonin abnormalities: A selective overview for the implications of suicide prevention. Eur. Arch. Psychiatry Clin. Neurosci..

[60] Heim C., Nemeroff C.B. (2001). The role of childhood trauma in the neurobiology of mood and anxiety disorders: Preclinical and clinical studies. Biol. Psychiatry.

